# Validity and reliability of the EQ-5D-3L™ among a paediatric injury population

**DOI:** 10.1186/1477-7525-11-157

**Published:** 2013-09-17

**Authors:** Mariana Brussoni, Sami Kruse, Kerry Walker

**Affiliations:** 1 British Columbia Injury Research & Prevention Unit, F511 – 4480 Oak Street, Vancouver, British Columbia, V6H 3 V4, Canada; 2 Child & Family Research Institute, Vancouver, British Columbia, Canada; 3 Department of Pediatrics, University of British Columbia, Vancouver, British Columbia, Canada; 4 School of Population & Public Health, University of British Columbia, Vancouver, British Columbia, Canada; 5 British Columbia Children’s Hospital, Vancouver, British Columbia, Canada; 6 University of British Columbia, Vancouver Fraser Medical Program, Vancouver, British Columbia, Canada

**Keywords:** Child, Trauma, Quality of life, Instrument, Measurement

## Abstract

**Background:**

Injuries are a leading cause of death and disabilities for children and youth globally. Measuring the health related quality of life of injured children and youth can help gain understanding of the impact of injuries on this population; however, psychometric evaluation of health related quality of life tools among this population is lacking. The purpose of this study was to determine the construct validity of the EQ-5D-3L™ for use among a population of injured young people and to examine the reliability of different modes of administration including paper and pencil, online and telephone.

**Methods:**

In total, 345 participants (aged 0 – 16) were recruited from a paediatric hospital in a large urban centre in British Columbia, Canada. To capture a variety of injury types and severity, patients were recruited from in-patient units and the emergency department. Data were collected at the time of recruitment and at one month post injury.

**Results:**

Repeated measures analysis (rANOVA) showed that EQ-5D-3L™ scores were different before and after injury and significant between group differences (Visual Analog Scale: *F* = 4.61, *p* = 0.011; Descriptive Scale: *F* = 29.58, *p* < 0.001), within group differences (Visual Analog Scale: *F* = 60.02, *p* < 0.001; Descriptive Scale: *F* = 92.37, *p* < 0.001), and interaction between variables (Visual Analog Scale: *F* = 10.89, *p* < 0.001; Descriptive Scale: *F* = 19.25, *p* < 0.001) were detected, indicating its suitability for assessment of post-injury health related quality of life. Bland-Altman plots confirmed that few differences existed between modes of administration.

**Conclusion:**

The EQ-5D-3L™ is an appropriate instrument for collecting health related quality of life data among injured children and can be administered via paper-pencil, online or by telephone.

## Background

Injuries account for nearly 40% of deaths in children ages 1 to 14 around the world [1,2] and are a leading cause of death and disabilities for children and youth in Canada [3]. Recent data indicate injuries resulted in 8.56 and 418.2 per 100,000 population death and hospitalization rates, respectively, for Canadian children and youth [3,4]. Paediatric injuries significantly impact quality of life across multiple domains, including physical, emotional and psychosocial health, and early identification of impairments assists with improving children’s outcomes [5]. Despite the substantial burden that injury represents for children and youth, research on the long-term impact of these events is scarce and what little is available can be challenging to interpret due to heterogeneity of study methods and measurement techniques. The need for standardized methods of comprehensive on-going data collection and interpretation regarding the outcome of these injuries persists [6-10].

Systematic measurement of post-injury health status in paediatric populations has the potential to impact prognostication from baseline presentation and can be used to assess recovery. This is valuable for the patient, caregiver, physician and healthcare administration. Addressing paediatric health-related quality of life outcomes is associated with improved long term health status as well as reduced health care costs [11]. With greater awareness regarding the magnitude and long term outcomes of child injuries, physicians can attend to previously overlooked areas and consider prevention possibilities. Health outcome data can also be extremely valuable in the evaluation of the monetary cost associated with injury and recovery. Currently, cost associated with paediatric trauma can be estimated from the Paediatric Trauma Score, a clinical scoring system devised to assess children’s vulnerability to traumatic injury [12,13], but an equivalent method applicable to non-traumatic injuries has yet to be devised [14]. The Injury Severity Score is a clinically based anatomical scoring system that assesses the severity of trauma involving multiple injuries [15]. While this tool applies to both more and less severe injuries [16], it has not been found to result in accurate cost predictions [14].

Health-related quality of life data have typically been collected via paper and pencil [17] or by telephone [18]. In recent years, the internet has gained popularity as an efficient and cost-effective tool for data collection and has been shown to be valid and reliable for a variety of instruments [19,20]. Study participants, especially younger demographics, also indicate preferences for this form of data collection [21,22]. To ensure efficiency and flexibility in data collection modes, and to reach a variety of audiences and maximize response rates, it is important to determine whether paper and pencil, telephone and online administration result in comparable data.

The EQ-5D-3L™ is a generic standardized measure of health status for an array of health conditions, treatments, and populations. The EQ-5D-3L™ has been used in the paediatric injury population in previous research due to its ease of application to a diverse group of injuries and broad coverage of applicable health domains [7]. Beyond its ability to provide information on injury outcomes, the tool has been suggested for use in the economic evaluation of trauma care [23]. Quality Adjusted Life Years (QALYs) can be derived from the EQ-5D-3L™ and are increasingly used to estimate costs associated with an injury [24-27].

The EQ-5D-3L™ includes two parts: the EQ-5D-3L™ descriptive system and the EQ-5D-3L™ visual analogue scale (VAS). The descriptive system consists of five dimensions including mobility, self-care, usual activities, pain/discomfort and anxiety/depression. The EQ-5D-3L™ is the original version of the instrument and allows respondents to rate each dimension using the following choices: ‘no problems’, ‘some problems’, and ‘extreme problems.’ Unique health states are calculated based on responses to all five dimensions and then converted to a single summary index (ranging from 1 for full health to 0 for death) using the time trade-off valuation technique provided by the instrument developers [28,29]. The VAS is presented in a thermometer-like fashion and records the self-rated health of a participant on scale from ‘best imaginable health state’ (100) to ‘worst imaginable health state’ (0) [28,29]. A line is drawn from a box in the centre of the page to a place on the thermometer-like figure to depict a current health state. Previous research has validated the EQ-5D-3L™ for use among adults in a general population [30] and children over 5 in a disease specific population [31]. The tool has been recommended as a post-injury assessment tool [10,32]; however, evaluation of its use on a pediatric injury population and with different forms of administration is limited [8,33].

This study evaluates the use of EQ-5D-3L™ for injured children and the reliability of different modes of administration of the instrument including paper and pencil, online and telephone.

## Methods

### Study sample and setting

Participants were recruited from the emergency department and medical units of a paediatric hospital in British Columbia, Canada. The patient and their parent or caregiver were approached regarding participation. Patients eligible for participation were identified in the emergency department at the time of triage and through review of the daily admission census for injuries admitted to the medical units. Eligibility criteria ensured that the child was 0 to16 years of age and had a primary injury diagnosis for which he or she was seeking treatment, the parent or primary caregiver and child (aged five years and up) were able to speak English, and the family resided in the province of British Columbia. Potential participants were provided with both verbal and written information about the study from a research assistant. Written consent and assent for participation in the study were obtained from parents or caregivers and children aged seven and over, respectively. Ethics approval for all study procedures was obtained from the Children’s and Women’s Health Centre of British Columbia Research Ethics Board and Public Health Agency of Canada Research Ethics Board.

### Data collection

Self-report of children’s health-related quality of life (HRQL) is considered the gold standard [17,34]. Some research suggests that self-report can be used for children ages 5–14, with parent proxies providing data when the child is unable or impaired without significantly compromising the quality of the data [35-37]. Wherever possible, child self-report EQ-5D-3L™ data were collected from enrolled children in addition to caregiver completion, resulting in two data sets for those children who also provided the self-reported information. To prevent redundancy and to ensure the use of current standards for paediatric data collection [36,37], child self-reported information was used when available for statistical analyses. In cases without child self-report, either due to the participant being under the age of 5, or unable to respond due to the nature or magnitude of the injury, parental proxy information was used [35].

The baseline questionnaire package collected demographic information, details regarding the injury event and the EQ-5D-3L™. Participants were instructed to report on their HRQL on the day prior to their injury. At one month post-injury, participants were mailed a hard copy of the EQ-5D-3L™ to complete reflecting on their current health status and return via a self addressed postage paid return envelope. To maximize response rates, participants were reminded about the study via telephone and email one week after follow-up packages were mailed out.

At both time points, parents as well as children aged 5 years and older completed the EQ-5D-3L™ VAS. Parents whose child was above 24 months of age as well as all children aged 13 years and older completed the EQ-5D-3L™ descriptive system.

To examine the equivalency of multiple administration modalities, a sub-sample of participants completed the same assessment three times in one day: Once by paper and pencil, once by computer and once via telephone. The order of modality completion was randomized to minimize order effects on participant responses.

### Statistical analyses

Data were analyzed using SPSS 20.0 software [38]. The conversion of the ED-5D-3L™ descriptive scale into a summary index was done by applying a formula based on the health state of a general population to the data collected for this study. Currently, no Canadian population value sets are available and, as such, the scoring algorithm was based on United Kingdom population level value sets [29].

### Construct validity

The EQ-5D-3L™’s ability to measure HRQL changes within a population of injured children was examined by exploring the relationship between pre-injury and post-injury responses. Previous paediatric injury research has used time spent in hospital as a measure for injury severity, with longer hospital stays indicating more acute injuries [39]. The use of length of stay as a surrogate measure has been validated in paediatric populations where the likelihood of medical fragility and co-morbidities are low [40]. To examine the relationship between HRQL and severity of injury, length of stay was stratified into three categories for the analyses: not admitted, admitted for one to three days, and admitted for four or more days. Patients not admitted represent less severe injuries, those admitted for one to three days represent moderately severe injuries and those admitted for four days or more represent more severe injuries.

Repeated measures analysis of variance (rANOVA) was performed for the descriptive scale as well as the VAS to assess whether the EQ-5D-3L™ discriminated between pre- and post-injury responses while testing for interactions with time spent in hospital. Post-hoc analyses examined differences across groups of injury severity. To account for multiple comparisons made when assessing the level of differences between categories of length of stay, the Bonferroni correction [41] was applied to the post-hoc analysis, and p-values were divided by the number of comparisons made.

### Reliability of modalities of administration

The Bland-Altman method [42] was used to assess the reliability of different modes of administering both the EQ-5D-3L™ VAS and the EQ-5D-3L™ descriptive scale. Data were initially examined through a series of simple plots. Total difference scores were computed for each mode of delivery for the EQ-5D-3L™ VAS and descriptive system separately. Average mean difference scores for each pair of modalities were calculated and compared two methods at a time via a series of scatter plots (i.e., paper and pencil compared with online; online compared with telephone; and telephone compared with paper and pencil). Agreement was evaluated by observing the dispersion of the differences and assessing the number of points beyond two standard deviations of the mean.

## Results

### Study sample

At baseline, 345 participants completed the questionnaire package and, of these, 253 provided complete one-month follow-up packages (150/59% included child self-report). Chi-square tests confirmed no significant differences in age, gender, and injury severity between those who completed both time points versus those who completed only baseline. Data on the validity of the EQ-5D-3L™ VAS and descriptive scale were available from 250 and 232 participants, respectively and a total of 44 participants completed the reliability testing of the questionnaire. Demographic details of the sample for both study arms are provided in Table 1.

**Table 1:** Participant demographics

		**Construct validity**	**Reliability of modalities**
		**N = 253**	**N = 44**
Treatment	Emergency Department	191	0
	Admitted 1–3 days	30	21
	Admitted 4+ days	32	22
	Unknown	0	1
Age of injured child*	0-2 years	27	6
	2-4 years	57	9
	5-7 years	46	5
	8-12 years	68	11
	13-16 years	54	14
Gender of injured child†	Boy	155	26
	Girl	96	18
Injury type**	Head Injury	44	3
	Upper Extremities Fracture	52	5
	Lower Extremities Fracture	22	6
	Dislocation, Sprain and Strain	25	3
	Minor External	39	1
	Internal Organ	2	1
	Facial	4	0
	Multiple Injuries	1	0
	Other‡	40	25

*missing = 1;

† missing = 2;

**missing = 24;

‡includes open wounds, burns, injury to nerves, dental injury, poisoning, and asphyxia.

### Construct validity

**Figure 1:**
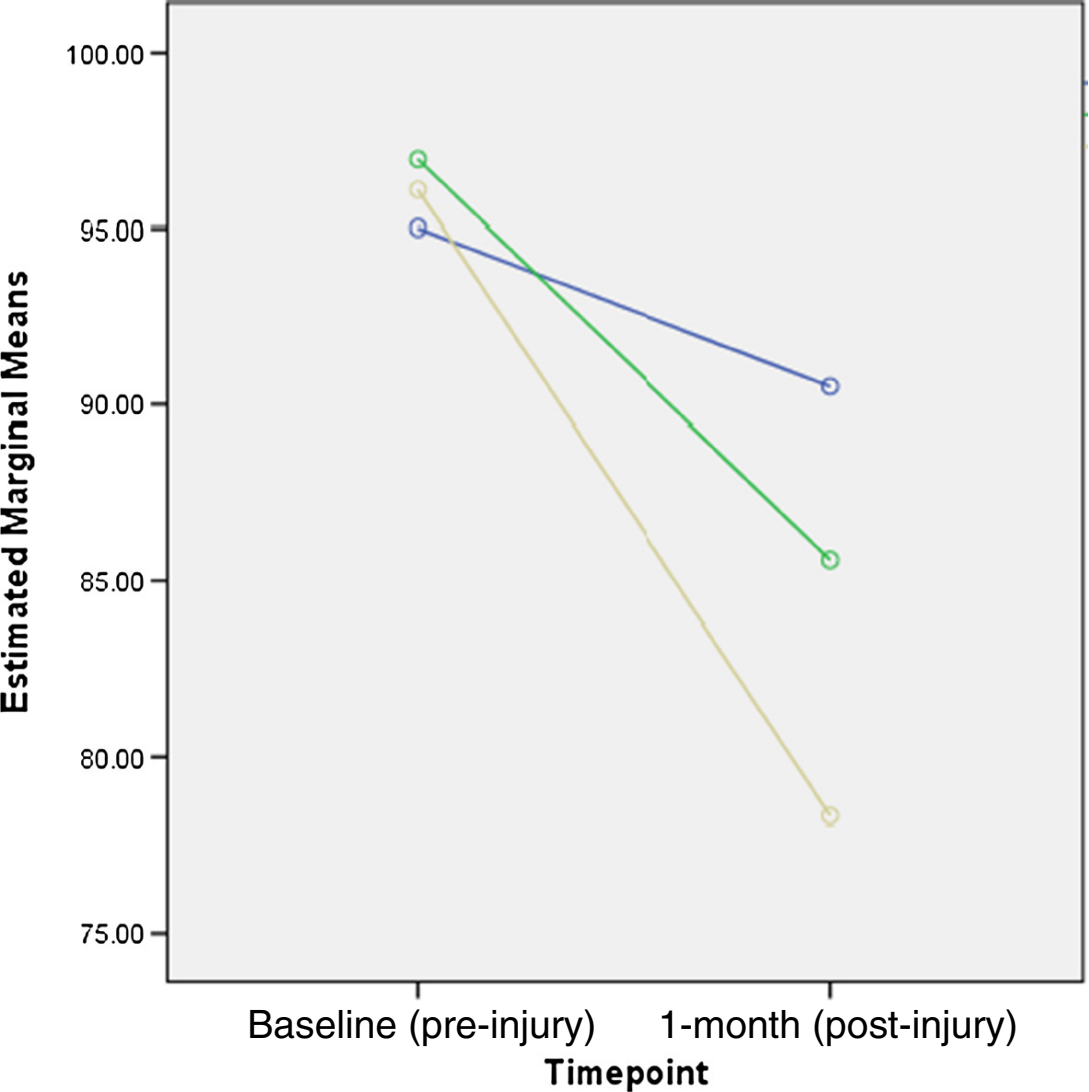
**Estimated marginal means of EQ-5D-3 L VAS scores at baseline and 1 month post injury.** Figure legend, Length of stay: “Blue circle symbol”: not admitted; “Green circle symbol”: 1–3 days in hospital; “Dark yellow circle symbol”: 4 or more days in hospital.

**Figure 2:**
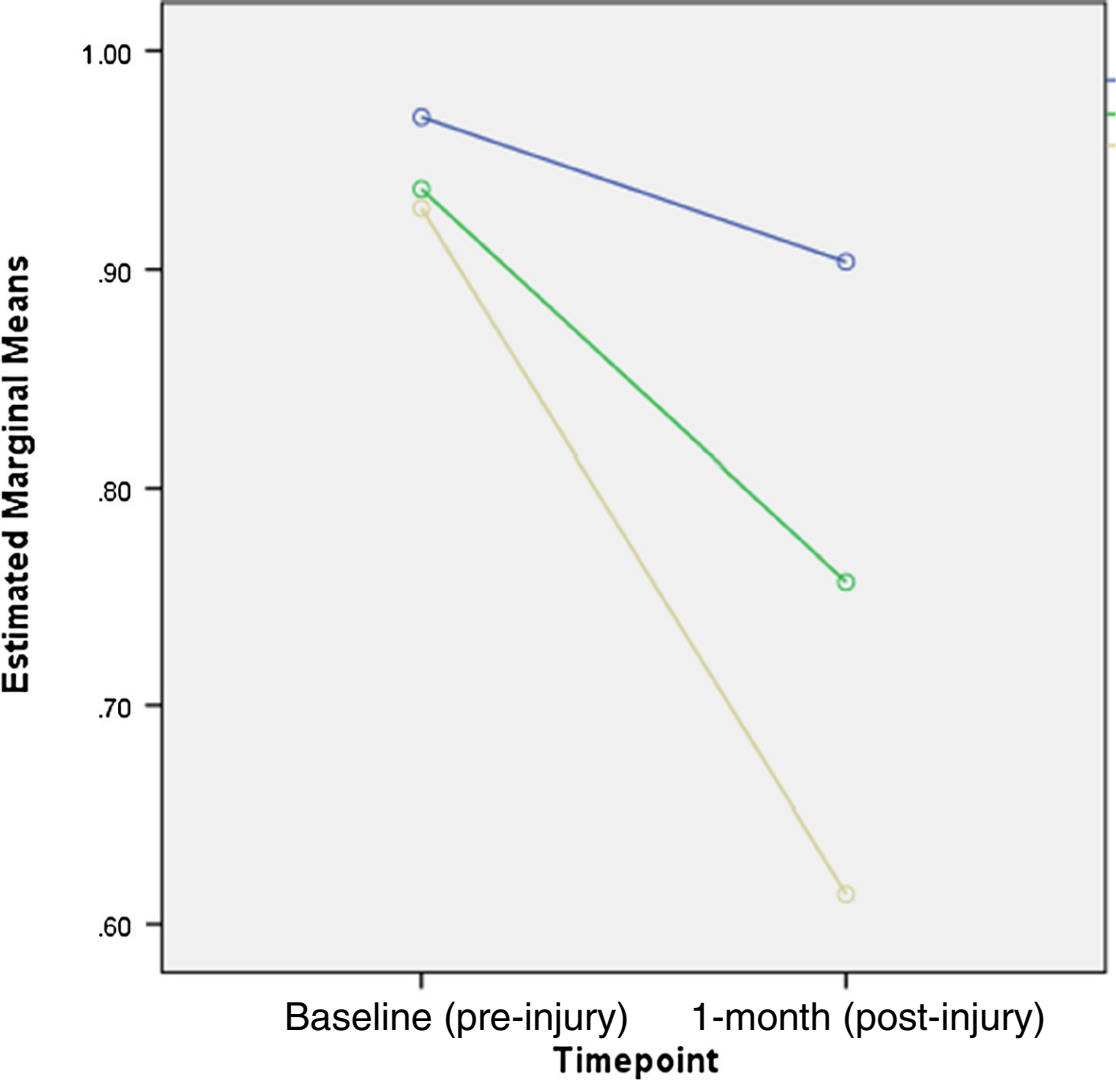
**Estimated marginal means of EQ-5D-3 L descriptive scale scores at baseline and 1 month post injury.** Figure legend, Length of stay: “Blue circle symbol”: not admitted; “Green circle symbol”: 1–3 days in hospital; “Dark yellow circle symbol”: 4 or more days in hospital.

Repeated measures ANOVA were conducted on EQ-5D-3 L ™ VAS scores and descriptive system scores. Scores from each category of length of stay were not significantly different at baseline, indicating comparable pre-injury health status. At follow up, all three categories of length of stay in hospital had significantly lower means for both the EQ-5D ™ VAS and the EQ-5D ™ index scores when compared to baseline (Table 2). Table 3 provides the results of the rANOVA and shows significant differences in one-month scores between categories of length of stay in hospital as well as differences in pre and post responses for both the VAS and the descriptive scale. Figures 1 and 2 illustrate the estimated marginal means both pre- and post-injury for the VAS and the descriptive score, respectively.

**Table 2:** EQ-5D-3L™Summary Scores at Baseline and one month post injury

**EQ-5D-3 L VAS**	**Total N = 250**	**Baseline mean (95% CI)**	**One month follow-up mean (95% CI)**	**Mean difference (95% CI)**
Not admitted	188	95.01 (93.79, 96.23)	90.50 (88.20, 92.80)	4.51 (1.91, 7.11)
Admitted 1–3 days	30	96.95 (95.08, 98.82)	85.58 (79.73, 91.43)	11.37 (5.22, 17.52)
Admitted 4+ days	32	96.09 (93.73, 98.45)	78.33 (73.10, 83.56)	17.76 (12.02, 23.20)
**EQ-5D-3 L Descriptive Scale**	**Total N = 232**	**Baseline mean (95% CI)**	**One Month Follow-Up mean (95% CI)**	**Mean Difference (95% CI)**
Not admitted	174	0.97 (0.96,0 .98)	0.90 (0.88, 0.93)	0.07 (0.04, 0.09)
Admitted 1–3 days	27	0.94 (0.88, 1.00)	0.76 (0.68, 0.84)	0.18 (0.08, 0.28)
Admitted 4+ days	31	0.93 (0.86, 0.99)	0.61 (0.51, 0.72)	0.31 (0.26, 0.36)

**Table 3:** rANOVA results: between and within group interaction

	**EQ-5D-3 L VAS**		**EQ-5D-3 L descriptive scale**	
	**F - Value**	**P - Value**	**F - Value**	**P - Value**
Between groups effects	4.61	.011	29.58	<0.000
Within groups effects	60.02	<0.000	92.37	<0.000
Interaction	10.89	<0.000	19.25	<0.000

For both the EQ-5D-3L™ VAS and descriptive system, the greatest drop when comparing pre- and post-injury score was observed among children who spent four days or more in hospital. The post hoc analysis showed statistically significant differences when comparing the VAS score of those not admitted with those admitted for 4 days or more (*p* value < .05). Comparisons of the descriptive scale showed significant differences between those not admitted to those admitted for 1–3 days (*p* value < .001) as well as those not admitted to those admitted for four days or more (*p* value < .001). Differences between those admitted for shorter stays (1–3 days) and longer stays (4 days or more) were also significant ( *p* value < .05).

### Reliability

**Figure 3:**
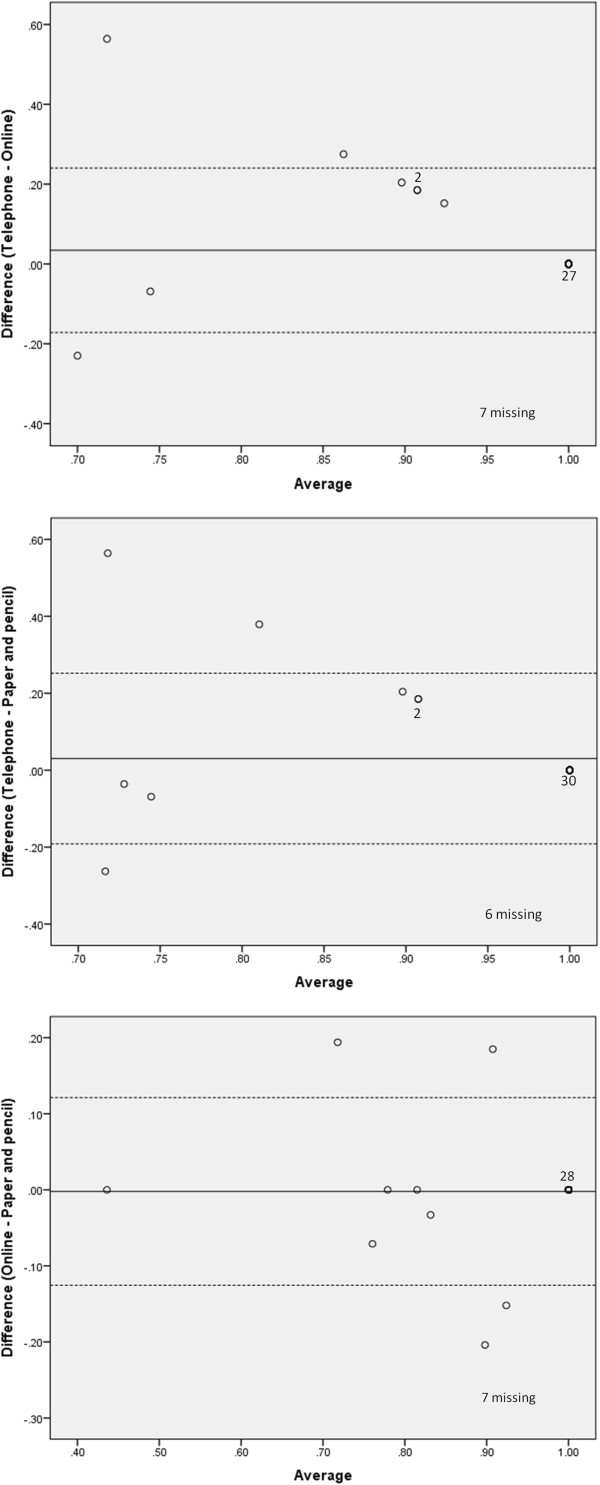
**Bland-Altman plots for EQ-5D-3 L descriptive scale scores.** Figure legend: …………… 95% confidence limits of the mean difference; _______mean difference. Note: The number of data points represented has been labeled for points >1.

**Figure 4:**
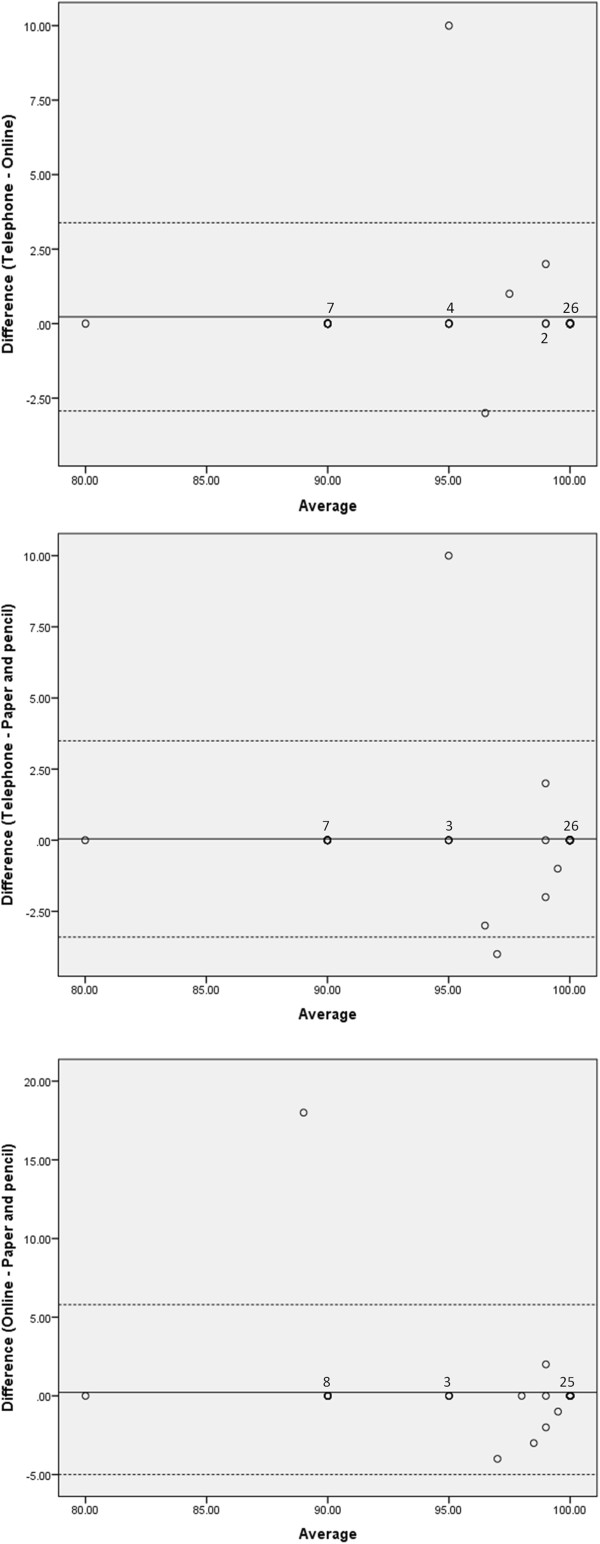
**Bland-Altman plots for EQ-5D-3 L VAS.** Figure legend: …………… 95% confidence limits of the mean difference; _______mean difference. Note: The number of data points represented has been labeled for points >1.

The comparisons of mean difference scores illustrated in the Bland-Altman plots show considerable consistency across the different modalities of questionnaire administration for both the EQ-5D-3L™ VAS and the descriptive system. Results for both measures show very few data points beyond two standard deviations of the mean when comparing the mean difference scores for each of the modalities. The analysis of the EQ-5D-3L™ descriptive system showed no more than four points beyond two standard deviations of the mean with the most variance exhibited between online and paper and pencil (Figure 3). The comparison of the VAS scale showed as few as two points beyond two standard deviations of the mean. For this measure the greatest variance was seen when comparing telephone with paper and pencil (Figure 4). When asked about preferred method of completion, 54% favoured online administration, 26% paper and pencil and 20% phone administration.

## Discussion

The EQ-5D-3L™ has been recommended for injury outcome studies [43]; however, understanding of its utility among paediatric populations is limited [8,33]. In the context of injury prevention, much of the existing research involving the administration of the EQ-5D-3L™ has been done with adult populations and is specific to a mechanism of injury or to particular types of injury [32]. Few studies have examined the use of the EQ-5D-3L™ among children [27,32], particularly comparing injuries of different levels of severity [44]. The purpose of this study was to assess the utility the EQ-5D-3L™ as a measure of HRQL for injured children ages 0 to 16 and determine if the EQ-5D-3L™ could be administered and interpreted reliably in varied modes of administration.

Our results indicate significant differences in the responses to both the EQ-5D-3L™ descriptive score and VAS score between baseline pre-injury status, and one-month post-injury indicating that both measures sensitively differentiate injured from non-injured states in the paediatric population. The EQ-5D-3L™ has been found to be reliable for comparisons of responses among injured adults [26,45] and has been identified as a useful measure for HRQL of injured adults [46]. Our study supports the reliability of the EQ-5D-3L™ for assessment of HRQL in injured paediatric populations presenting in the emergency department and inpatient units.

Administering the EQ-5D-3L™ descriptive system and VAS to children via paper and pencil, online, or the telephone resulted in sufficiently consistent responses. To our knowledge, the existing research on EQ-5D-3L™ administration has compared only two modes of administration with one another [22,47,48]. Moreover, previous research has been inconclusive, with some confirming our findings regarding the equivalency of different administration methods [48-51], and others finding differences between modes [47,52]. Our study broadens the valid modes of administration of the EQ-5D-3L™ for injured children and implies the potential use in other areas of paediatric care. The use of multiple modes of administration in paediatric health measures has been shown to be valid [21]. This permits flexibility when administering instruments to allow for variation in participant capabilities and response method preference, as well as study budgets and timelines [47].

While this study supports the application of the EQ-5D-3L™ for injured children, some methodological challenges do exist. This study uses a combination of self-report and parent proxy report. Although, parent proxy has been recognized as a viable option for child health status [35-37], some research suggests there are discrepancies in parent and child responses. For example, one study on the use of parental proxy data among injured children found that children tended to rate their HRQL significantly higher than the ratings of their parents in the short term, while in the long term the ratings converged [34]. Future studies involving the EQ-5D-3L™ and HRQL data should consider the possibility of this effect in study design as well as interpretation of results. Furthermore, it is possible that responses to alternate modes of instrument administration vary at subsequent post-injury follow-ups and this issue would benefit from future investigation.

There is the potential for bias in recalling pre-injury HRQL; however, this is the recommended method for measuring pre-injury (baseline) status, rather than referencing against age- and sex-matched population norms [53]. This is because injured populations rate their pre-injury HRQL status as significantly higher than matched population norms, possibly because they are more physically active than the general population [53].

Participants who completed the instrument via multiple modalities did so over the span of one day. While this is a short time frame and may have increased recall and thus the correlation between measures, the nature of injury and recuperation means that rapid changes in quality of life and health status can occur from one day to the next. In the interest of limiting measurement differences due to actual quality of life changes, we chose to undertake data collection within the span of one day. Previous research indicates that recall bias can be minimized with the use of high quality questionnaires administered to participants and proxies who are unaware of the study hypothesis [54]. While the participants were not blinded to our hypothesis in our study, we used a standardized measure for both children and proxies. The fact that agreement among different modalities was not perfect could indicate that recall bias was minimized in our study.

## Conclusion

Childhood and youth injury continues to be a leading cause of morbidity and mortality globally, yet there is a paucity of understanding regarding their effects on HRQL. To provide comprehensive care, monitor outcomes and assess the economic and societal burden of injuries, it is essential that this area of research be expanded. To this end, the results of our study are important in encouraging this research. Our results verify that the EQ-5D-3L™ descriptive score and VAS can be utilized in the paediatric injured population for collecting HRQL data, whether self-reported or proxy reported, and that these data can be validly collected using paper-pencil, online or telephone modes of administration.

## Competing interests

The authors declare that they have no competing interests.

## Authors’ contributions

MB led the design and conception of the study, participated in the interpretation of the data and provided critical intellectual input to the development of the manuscript. SK participated in the study design, carried out the data analysis and drafted the manuscript. KW led participant recruitment and assisted with the draft and revisions of the manuscript. All authors read and approved the final manuscript.

## Authors’ information

MB (PhD) is an assistant professor in the Department of Pediatrics and the School of Population and Public Health at the University of British Columbia. SK (MPH) is a Social Science Researcher at the BC Injury Research and Prevention Unit. KW is a student at the University of British Columbia in the Vancouver Fraser Medical Program.
